# Editorial: Soil-microbial interactions

**DOI:** 10.3389/fmicb.2023.1213834

**Published:** 2023-05-16

**Authors:** Phesheya Dlamini, Lerato M. Sekhohola-Dlamini, A. Keith Cowan

**Affiliations:** ^1^Department of Plant Production, Soil Science and Agricultural Engineering, University of Limpopo, Sovenga, South Africa; ^2^Institute for Environmental Biotechnology, Rhodes University, Makhanda, South Africa

**Keywords:** soil, rhizosphere, phylogenetic analysis, microbiome, plant-soil-microbe interactions

Recent perspectives from various panels of the Food and Agriculture Organization (FAO) of the United Nations (UN), in particular the Intergovernmental Technical Panel on Soils (ITPS) and the United Nations Environment Programme (UNEP), have reiterated that soil, particularly arable soil, the covering that facilitates ecosystem services critical to sustaining life, and the majority of soil resources are at best in only fair, poor or very poor condition (FAO and ITPS, 2015; FAO and UNEP, 2021). These reports emphasize the importance of regular soil function assessment to determine overall soil health at a regional and global level.

Soil is a complex microhabitat that comprises mineral particles of different sizes, shapes and chemical characteristics, together with soil biota and organic compounds in various stages of decomposition (Daniel, 2005). The soil minerals present a biogeochemical interface, where organic and inorganic constituents of the soil interact (Totsche et al., 2010). Specifically, the surfaces of soil aggregates and the complex pore spaces between and inside the aggregates provide microhabitats for soil microorganisms. The complex and variable soil matrix harbors a consortium of organisms that strongly influence its biogeochemistry by forming and decomposing soil organic matter, the planet's largest terrestrial stock of organic carbon and nitrogen, and a primary source of other crucial macro and micro-nutrients (Crowther et al., 2019). Indeed, microorganisms inhabit diverse geological environments and create environments conducive to themselves and other life forms. Thus, it is pertinent to understand the relationship between microbial diversity and soil functionality, more especially considering that 80–90% of the processes in soil are reactions mediated by the microorganisms (Nannipieri et al., 2017). The subterranean microbiome is also linked to the aboveground biomass via the rhizosphere and is critical for sustainability in both natural (Coban et al., 2022; Hua et al., 2022; Vetterlein et al., 2022) and previously disturbed and/or degraded but restored ecosystems (Sekhohola-Dlamini et al., 2022). Also, cover vegetation, cultivated either as a single crop or as a mixed crop, provides several ecosystem services that help achieve many of the UN's sustainable development goals (SDGs) (Lamichhane and Alletto, 2022). Needless-to-say, there is a need for thorough mechanistic understanding of microbial interactions with each other and with soil properties, which can be achieved through in-depth experimental and computational methodologies (Tang, 2019).

Because of their significant contribution, microbial interactions within the geosphere lie at the heart of the interdisciplinary field of soil biogenesis, quality and chemical and physical characteristics. Consequently, successful restoration of ecosystem functions, and enhanced soil health and quality can only be possible through thorough understanding of microbial interactions with each other, the soil, their associated plant communities, and the impacts these dynamics have on the underlying molecular and biogeochemical functions (Sullivan and Gadd, 2018). Extensive research demystifying plant-soil-microbe interactions highlights insights into beneficial soil ecosystem functions, which are widely explored in soil remediation and land rehabilitation as well as improved food production. For instance, the functional dynamics in the rhizosphere; a biologically active zone where complex interactions among plant roots, soil and microbes occur, play a vital role in driving vegetation cover and soil restoration (Liu et al., 2019; Villarino et al., 2021). Studies have correlated microbial sequencing datasets to physico-chemical parameters to infer and contextualize soil community interactions. By developing theoretical frameworks that elucidate the multi trophic interactions found in different soils, conceptual models that seek to decipher soil microbial functional guilds have emerged (Singh et al., 2014; Levy-Booth et al., 2019; Hicks et al., 2022). While chemical and physical characteristics change slowly from year to year, soil biology is dynamic, with implications for soil physico-chemistry.

Extensive phylogenetic identification of microbial populations and their potential environmental functioning has revealed previously unrecognized ecological interactions between biological entities and the geosphere. To this end, Zhang S. et al. highlight potential ecological mechanisms underlying microbial population structure-function associations in soil aggregates to emphasize the assembly of aggregate microbes as an indicator of the interactions between agricultural soils and microbial communities. Bacterial quorum-sensing (QS) is a primary means of allowing communication between cells or populations, is cell-density dependent, and enables coordinated response mechanisms to manifest. Using iTRAQ, a shotgun-based quantitation method, which allows for concurrent identification and quantification of proteins in different samples within a single experiment, Zhang and Lyu demonstrated inhibition by quorum-quenching lactonase (YtnP) from the consort species *Bacillus pumilus* of metabolic signaling in *Ketogulonicigenium vulgare* in the fermentative production of the ascorbic acid precursor, 2-keto-L-gluonic acid (2-KLG). Continuous cropping of soils degrades both soil organic matter and soil structure. Miao et al. confirmed that ginsenosides, a group of *Panax quinquefolius*-derived steroid-like saponins, significantly contribute to soil deterioration and increase the abundance of pathogenic fungi in the continuous cropping of this herb. The study by Sui et al. shows that along with soil physicochemical parameters, the composition and diversity of fungi in the rhizosphere of the Alpine grass *Deyeuxia angustifolia* decreased with increasing altitude. Soil nitrate-nitrogen (NO_3_-N), moisture content and pH were closely linked to species richness and phylogenetic diversity, indicating the sensitivity of soil-microbe interactions and as key in determining fungal community diversity. Zhang Z. et al. also explored soil-microbe interaction sensitivity by examining the effect of lead contamination on microorganisms in tea gardens to determine how this contaminant impacts essential soil microorganism function. The predicted main function of the bacterial community was amino acid transport and metabolism, while the fungal community's trophic mode was mainly pathotroph-saprotroph. They show that lead concentration was the factor that most strongly affected soil bacterial and fungal community structures, with the latter more affected than the former.

Contributions to this Research Topic used cultivated and natural ecosystems to explore soil-microbe interactions. Most used a metagenomics approach to elucidate community structure and species diversity. In one instance, the functional abundance of soil bacteria and fungi was examined by high-throughput sequencing. In another, iTRAQ-based proteomics analysis was used. All highlight the intimate and sensitive association between microbes and the soil. We hope this collection encourages further research toward the applications of microbes to ensure sustainable soil processes, including fundamental biotic responses to evolving geosphere environments, such as bioremediation of disturbed terrain and soil fertility restoration.

## Author contributions

All authors drafted, read, edited, and approved the manuscript for publication.
